# Association of SARS-CoV-2 and Polypharmacy with Gut–Lung Axis: From Pathogenesis to Treatment

**DOI:** 10.1021/acsomega.2c02524

**Published:** 2022-09-16

**Authors:** Jonaid Ahmad Malik, Sakeel Ahmed, Zahid Yaseen, Muteb Alanazi, Tareq Nafea Alharby, Hisham Abdulaziz Alshammari, Sirajudheen Anwar

**Affiliations:** † Department of Pharmacology and Toxicology, National Institute of Pharmaceutical Education and Research, Guwahati, Assam 781101, India; ‡ Department of Biomedical Engineering, Indian Institute of Technology Rupnagar 140001, India; § Department of Pharmacology and Toxicology, National Institute of Pharmaceutical Education and Research, Ahmedabad, Gujarat 382355, India; ∥ Department of Pharmaceutical Biotechnology, Delhi Pharmaceutical Sciences and Research University, New Delhi, Delhi 110017, India; ⊥ Department of Clinical Pharmacy, College of Pharmacy, University of Hail, Hail 81422, Saudi Arabia; # Department of Pharmacology and Toxicology, College of Pharmacy, University of Hail, Hail 81422, Saudi Arabia

## Abstract

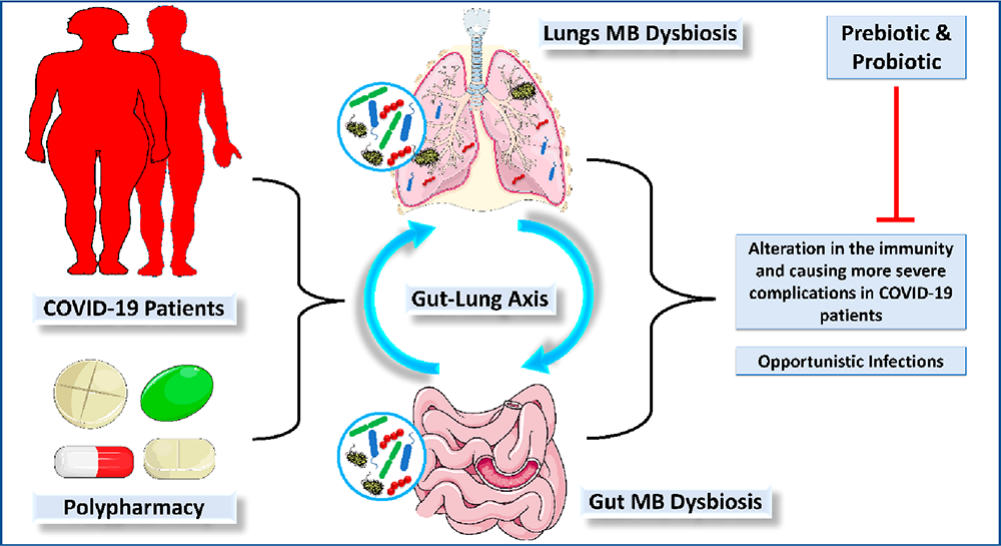

SARS-CoV-2 is a novel infectious contagion leading to COVID-19 disease. The virus has affected the lives of millions of people across the globe with a high mortality rate. It predominantly affects the lung (respiratory system), but it also affects other organs, including the cardiovascular, psychological, and gastrointestinal (GIT) systems. Moreover, elderly and comorbid patients with compromised organ functioning and pre-existing polypharmacy have worsened COVID-19-associated complications. Microbiota (MB) of the lung plays an important role in developing COVID-19. The extent of damage mainly depends on the predominance of opportunistic pathogens and, inversely, with the predominance of advantageous commensals. Changes in the gut MB are associated with a bidirectional shift in the interaction among the gut with a number of vital human organs, which leads to severe disease symptoms. This review focuses on dysbiosis in the gut–lung axis, COVID-19-induced worsening of comorbidities, and the influence of polypharmacy on MB.

## 1 Introduction

### 1.1 Lung Microbiota in a Disease

Microbiota (MB) is an umbrella term incorporating a complex collection of microbes, including viruses, bacteria, fungi, and parasites found on the body surfaces, that are directly in contact with the surroundings.^1^ MB plays a crucial role in homeostasis, including optimum metabolic functioning and proper immune response, and thereby, immune action influences the composition of the MB. Lately, extensive research on intestinal MB has revealed that the human intestine is colonized by approximately a hundred trillion microbes^2^ that contribute to physiology, metabolism, immunological functions, and nutrition. Dysbiosis of intestinal MB has been linked to various disease conditions, including gastrointestinal (GIT) disorders like inflammatory bowel disease (IBD) and obesity.^3^ The skin, upper respiratory tract (URT), and the genitourinary tract serve as a habitat for several species of MB and play an important role in the proper functioning of the immune system (site-specific immunity).^4,5^ Studies indicated that “the normal lungs are free from bacteria.″ However, the use of culture-independent techniques (CIT) indicate the presence of microbes in mammalian lungs.^6^

### 1.2 Physiology of Respiratory System and Origin of Lung MB

The respiratory system is mainly categorized into upper respiratory tract (URT) and lower respiratory tract (LRT). The nostrils, nasal cavities, pharynx, epiglottis, and larynx constitute the URT, whereas the trachea, bronchi, bronchioles, and lungs constitute the LRT. The lungs are primarily involved in exchanging gases, enabling the transfer of oxygen to the blood, and expelling carbon dioxide from the body.^7^ Recent advances in CIT for microbial estimation have indicated that the lungs are colonized by various MB communities, involving phyla *Firmicutes*, *Bacteroidetes*, *Proteobacteria*, *Actinobacteria*, *Fusobacteria*, and *Cyanobacteria*.^7^ Interestingly, studies suggest that healthy lungs are inhabited by various genera of *Prevotella, Streptococcus, Veillonella, Neisseria*, and *Haemophilus*.^8^

A pathogen’s virulence plays a crucial role in immunological response. The threshold of immunological response depends on parameters like environmental factors, genetic make-up, diet, stress, and age.^9−12^ A higher threshold of immunological response is required to prevent exaggerated inflammatory response in the lungs. For this purpose, the pulmonary system has several site-specific mechanisms to control inflammatory reactions, e.g., compared with macrophages in other body areas, alveolar macrophages are prevalent in the airway (>95%) and express lower levels of MHC Class II and costimulatory molecules. Also, alveolar macrophages display a suppressive phenotype because of the production of IL-10 and TGF-β.^13^ Airway epithelial cells yield elevated levels of TGF-β, IL-10, and GM-CSF to control DC responsiveness and alveolar macrophage activation.^14^ Toll-like receptors (TLR) and other pattern recognition receptors (PRRs) play a crucial role in stimulating the innate immunological responses followed by adaptive immunological responses. Studies indicate that TLR-4 molecules are expressed in alveolar and bronchial cells (mostly intracellular).^15^ With the help of CITs, studies have recently shown that the LRT, including the lungs, has been inhabited by various communities of MB, with the URT being an exception.^16^ Several research studies have shown that MB offers various health benefits by maintaining the immune system homeostasis.^17^ Investigation of lungs samples for the microbiome is very challenging considering the microbiome of the lung has low biomass and risk of contamination from oral and nasal areas during bronchoscopy.^1^ The procurement of samples up to the glottis using two bronchoscopes followed by a serial bronchoalveolar lavage and lower airway protected brush suppressed the possibility of contamination. It was concluded from the analysis of the composition of MB in oral wash, bronchoalveolar lavage fluid (BALF), nasal swab, and gastric aspirate samples that the respiratory tract is inhabited by a homogenous MB that decreases in biomass while going from the URT to the LRT. Also, the MB in the lungs is similar to the oral and nasal MB, which indicates that the MB in the lung might originate from the URT via breathing.^18^

## 2 Lung MB in the Regulation of Homeostasis

### 2.1 Promote the Turnover of the Lung Immune System

Various studies have highlighted the benefits of MB to the host, including maintaining the structure and functions of mucosa, improving adaptive and innate immunity, and providing protection from pathogenic infections.^19,20^ The Peyer’s patches, isolated lymphoid follicles, and mesenteric lymph nodes constitute the gut-associated lymphoid tissues and are not fully developed in germ-free mice.^3,21^ However, no evidence indicates that the MB of the lung had similar effects on the development of pulmonary mucosa-associated lymphoid tissue (MALT). Homeostasis of the intestine is maintained by PRRs detecting compounds having microbial action, which further leads to the differentiation of regulatory T cells (Treg) and Th17 cells. Similarly, lungs localized with PRRs could also detect compounds with microbial activity from lung MB and convert naive T cells into Th1 cells (but not Th2 cells).^22−25^

### 2.2 Inhibition of Increased Immunological Responses in Acute Infection

After the first 2 weeks of birth, microbial contamination is enriched in the lungs. Changes in the bacterial composition are seen as gamma *Proteobacteria* and *Firmicutes* shifting towards *Bacteroidetes*. Any variation in the composition of MB that colonizes in the lung is directly connected with the progression of Helios-negative Treg cells in the lungs in a PD-L1-dependent manner. The absence of MB or inhibition of PD-1 results from an exaggerated inflammatory response to allergens during childhood.^26^ The MB residing in the URT restricts lethal inflammation in the lungs caused by influenza in a TLR-2 and alveolar macrophages-dependent manner.^27^

## 3 Involvement of MB in Lung Disorders

The microbiome’s significance to intestinal health and various disorders has been extensively studied in disorders like IBD, ulcerative colitis (UC), and crohn’s disease.^28,29^ In the recent past, it was observed that the lung MB contributes to lung disorders and the extent of changes in the microbiome determines the risk of the disease, drug responses, and clinical outcomes.^30^ Multiple factors disrupt the lung MB: anatomical injuries, pathological effects, defects in the immune system causing chronic pulmonary disorders.^31^

### 3.1 Asthma

Asthma is a chronic multifactorial disease considered the consequence of genetic and environmental factors like allergens.^32^ The disease is highly prevalent in high-income nations, which indicates that the etiology of asthma is influenced by the living conditions because of the altered diverse composition of the MB inhabiting the lungs.^33^ It was observed that children exposed to a range of environmental microorganisms showed a lower risk of asthma.^34^ Various research on asthmatic patients identified a difference in the composition of MB compared to healthy subjects.^35,36^ The accurate explanation of this disease might be the more frequent *Proteobacteria* and less frequent *Bacteroidetes.* Therefore, the microbiome combination and the relationship between the lung MB and host plays a critical role in the etiology and development of asthma.^1^

### 3.2 Chronic Obstructive Pulmonary Disease (COPD)

COPD is a chronic inflammatory lung disease that causes obstructed airflow from the lungs.^37^ Compared with asthmatic patients where altered MB is seen,^36^ a study focusing on the microbiome’s contribution in COPD patients showed similarity in the microbiome composition between the lungs of mild to moderate COPD patients and healthy subjects.^38^ Alteration in the MB composition could only be detected in patients suffering from advanced COPD.^16^ Microorganisms like *Proteobacteria* or *Firmicutes* were more abundant and *Bacteroidetes* was less abundant,^39^ which are quite similar to the changes in the MB of asthmatic patients. Nevertheless, COPD and asthma are two different illnesses of the lung, which indicates that, apart from the changes in the lung microbiome, other factors collectively add to the disease that are more prominent than the involvement of MB.^1^

### 3.3 Cystic Fibrosis (CF)

CF is an inherited disorder affecting the pulmonary system, especially the lungs. The disorder is characterized by the progression of bronchiectasis and obstructive lung disease.^40^ The involvement of lung MB in the pathology of CF is not yet clearly understood. Respiratory pathogens like *Staphylococcus aureus* and *Pseudomonas aeruginosa* were present in the sputum obtained from young patients suffering from CF during clinical stability and exacerbations.^41^ Therefore, these exacerbations associated with CF are believed to be induced by bacterial infections. However, some studies indicate antibodies have no major influence on the progression of CF.^42,43^ Hence, exacerbation associated with CF is not a result of the increased bacterial load or reduced diversity. The association of the lung microbiome with the pathogenesis of CF is far more complex than it was believed before.^1^

## 4 Interkingdom Crosstalk

The interkingdom communication serves as the bridge between MB and the host in which the host produces small molecules such as hormones, and hormone-like molecules such as dynorphins produced by microbes serve as the medium to produce signaling. Many bacteria use the adrenaline/noradrenaline system to regulate and propagate virulence.^44^ The microorganisms use the host’s stress signals as a stimulating indication to release molecules that can produce various detrimental effects and play a crucial role in their survival and causing disease. A study showed *Salmonella typhi* sensing the host’s neuroendocrine stress hormones (adrenaline) and releasing hemolysin E (a toxin) lead to hemolysis of red blood cells, which was successfully halted by using β-adrenergic antagonist propranolol. In *Escherichia coli* O157:H7, adrenaline and noradrenaline signaling influenced bacterial virulence and motility. The specific membrane receptor (a membrane sensor QseC) has been found to express virulence genes in response to interkingdom cross-communication.^45^ Another experimental study shows an increase in virulence and invasive characteristics upon exposure of *Salmonella Typhimurium* to catecholamines when grown in *in vitro* conditions. The supernatants of *Salmonella typhimurium* were grown in the presence of catecholamines, which resulted in reduced porcine mitogen-induced lymphocyte proliferation. This indicates that stressful conditions modulate immune functions that can be explored further to create novel approaches to tackle microbial diseases.^46^

## 5 COVID-19 Shows Lung Fungal Dysbiosis

The lung mycobiome has been explored to study the pathological relation of fungi in causing diseases. Since it is a part of lung MB, the mycobiome is believed to be balanced in healthy people compared with patients with pulmonary disorders such as COVID-19. The lung mycobiome has been recognized as one of the components causing inflammation and modulating the immune response, thus suppressing lung function and disease progression.^47^ Recently, a study proposing fungal dysbiosis seen in COVID-19 patients has been related to the overgrowth of *Candida albicans*, while a few reports have seen COVID-19-associated pulmonary aspergillosis.^48^ According to another meta-analysis, *Candida* species like *Candida albicans* have been frequently isolated fungi present in severely affected COVID-19 patients.^49^ The serious imbalance of the mycobiome in lungs attributed to the wide use of antibiotics and corticosteroids, along with COVID-19 severity, may also lead to the invading of internal organs, thereby causing deep systemic infection.^50^ Compared with the bacterial role in the lung microbiome, the presence of fungi in healthy individuals may be less studied because of rare cases of this particular pathogen associated with COVID-19. Nevertheless, the role of mycobiome dysbiosis cannot be ruled out and is considered as one of the symptoms in severe cases of COVID-19.

### 5.1 MB–Virus Interaction

**Figure 1:**
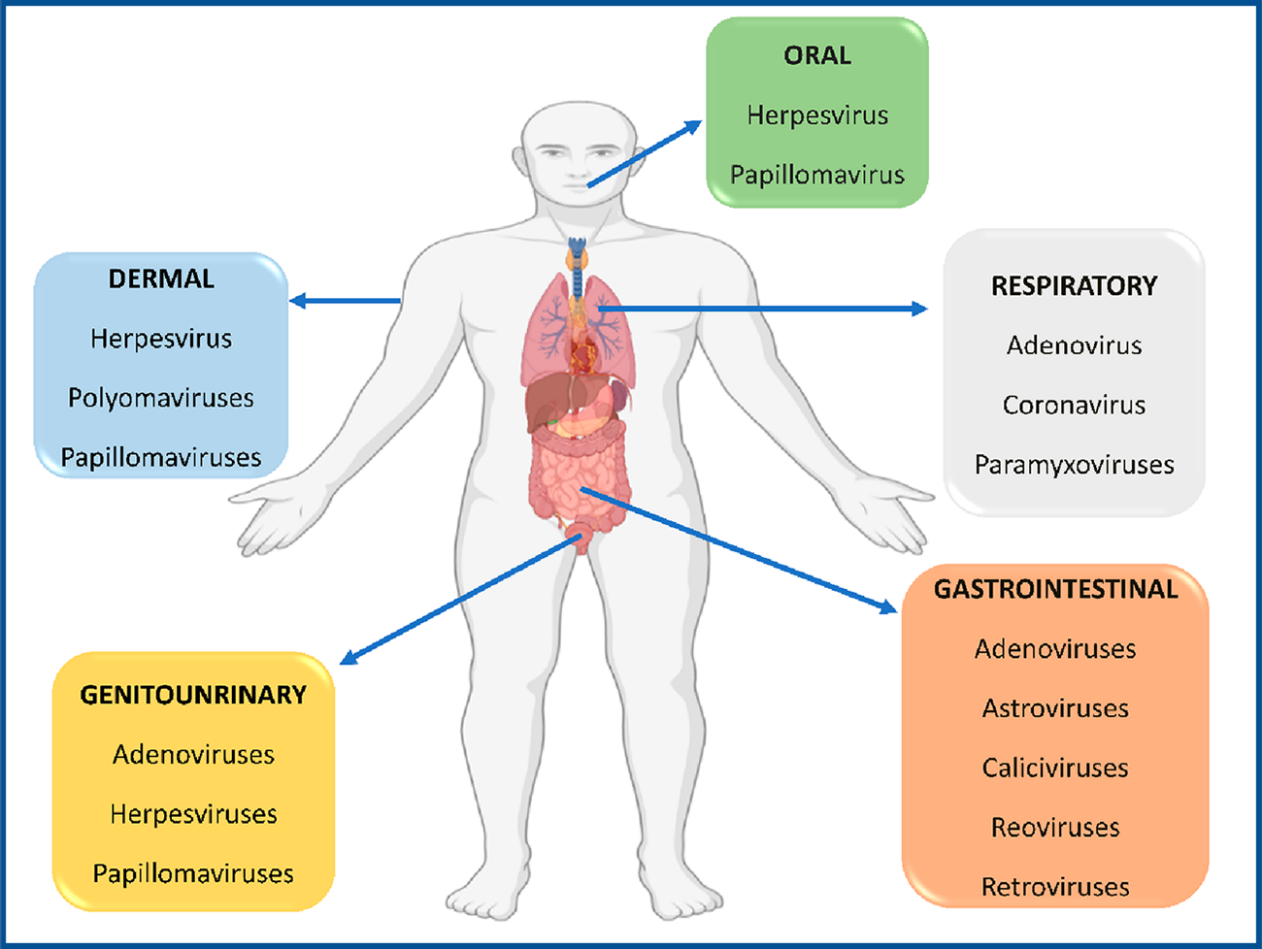
Viruses that are commonly found in the human body. MB–virus interaction, anatomical locations, and viral diversity display the most common viral species at each location. Viruses can infect and interact with the local microbial ecology at each location.

Viruses are a diverse collection of biological organisms that rely on host cell machinery to increase. Most viruses are recognized on the basis of their ability to cause disease and how they do so. Nonetheless, healthy people also carry nonpathogenic viral communities. The viral populations and diverse types of viruses associated with the human body are briefly shown in Figure 1.^51^ The coexistence of viruses and bacteria in the microbiome inspires researchers to look into viral evasion mechanisms that allow pathogens to be tolerated by the immune system.

MB-virus interactions have been studied using various laboratory models, the most popular of which are germ-free mice (GFM) or antibiotic-treated mice (ATM). GFM are born microbiologically sterile and raised in germ-free surroundings. Body weight is maintained by supplying GFM with 30% more calories/day than average animals, which demonstrates the relevance of the microbiome for nutrition.^52^ Despite being normally healthy and productive, they have immature immune systems, which makes experimental understanding difficult.^53^ However, GFM provide a specified environment free of microorganisms that can be colonized with one or more bacterial strains to study MB–virus interactions. As a result, GFM are an essential model for studying MB-virus interactions. ATM have also been used to examine MB–virus interactions since germ-free animals need specialized facilities and are costly to manufacture and maintain. The mice are given a cocktail of antibiotics in this paradigm, and knockdown is validated using culture-based approaches.^54^ The antibiotic treatment strategy has several drawbacks, including the possibility of altering host physiology and unspecifics caused by unculturable pathogens, partial knockdown, and antibiotic resistance.

In contrast, the antibiotic therapy is relatively inexpensive, can be employed with any mouse strain and other animal models, and allows animals to develop physiologically and immensely in the presence of MB before treatment. Although GFM and ATM models have advantages and disadvantages, it’s wise to utilize both when studying MB–virus interactions. Finally, to investigate the virus, it is necessary to use the natural route of infection. Oral inoculation is recommended for enteric viruses, intranasal inoculation is recommended for respiratory viruses, etc. Avoidance of the natural route of infection and, thus, the relevant microbial community can considerably impact the outcome.^55^

#### 5.1.1 MB-Virus Interaction in Humans

The microbiomes are essential for the host’s health. They also protect us from pathogens.^56^ When talking about viral infections, microbiomes can influence and are influenced by pathogenic viruses.^57^ Studies reveal that the gut of healthy humans harbors around 45% of mammalian viruses as a part of virome (virobiota) without any clinical symptoms. However, unlike bacteria, they can modulate the immune response.^58−60^ In a recent study, the metagenomic analysis revealed the phage diversity and their host range across isolated gut bacteria where the majority of phage viral clusters (VCs) were restricted to a single bacterial species, and some VCs are predicted to infect several species, genera, families, orders, and even classes. This study further demonstrated that phages in urban samples preferred to infect *Bacteroides* over *Prevotellaceae* bacteria. In contrast, the phages identified in rural samples (Peru, Tanzania, Madagascar, and Fiji) infect *Prevotellaceae* over *Bacteroides* bacteria. The highest number of VCs were isolated from *Lachnospira*, *Roseburia*, *Agathobacter*, *Prevotella*, and *Blautia* A. This same study revealed the link between phage viral clades with isolated gut microbiomes.^61^ A study by Tisza et al. revealed approximately two thousand phages are linked with common chronic diseases.^62^ Studies have demonstrated that a host’s microbiota can impact infections by various animal viruses.^63^ MB inhibits some infections, while some are promoted via direct or indirect effects on the host/virus. The viruses localized in the GI tract are most studied because it is the initial infection site, and a wide microbial community resides there.^64^ Bacteria isolated from human nasopharynxes, such as *Staphylococcus aureus, Haemophilus influenza, Pseudomonas species, Streptococcus pyrogen*, and *Streptococcus pneumonia*, increased the risk of death in influenza-affected adults and children.^65^ The microbiota’s impact on virus fitness is depicted in Table 1. The 16s RNA analysis of feces samples of 20 hospitalized children with acute viral gastroenteritis (AGE) revealed an increased *Campylobacteriaceae, Methylobacteriaceae*, *Neisseria* family, *Enterobacteriaceae,* and *Sphingomonas* family than the control group.^66^ The *Neisseria* family includes *Neisseria meningitides* and *Neisseria gonorrhea*, responsible for meningitis and gonorrhea.^67^ In contrast, *Campylobacter* causes bacterial diarrhea,^68^ which cannot be ignored. In COVID-19 patients, intestinal microbiomes are confirmed using the metagenomic sequencing analysis of fecal samples.^69^ At the time of hospitalization, the most common opportunistic pathogens (OPs) that are increased in COVID-19 patients include *Actinomyces viscous*, *Clostridium hathewayi*, and *Bacteroides nordii*. Moreover, gut dysbiosis endured even after negative tests for SARS-CoV-2.^70^ Overall, evidence suggests that COVID-19 patients had altered gut microbiomes and increased chances of opportunistic pathogens. Interestingly, the microbiomes can enhance the action of antiviral therapy,^71^ e.g., reduction in the efficacy of anti-HIV-1 drugs in women with vaginal microbiomes dysbiosis.^72^ This also supports the MB–virus interaction. Furthermore, there is a need of more metagenomic studies to reveal the further link between MB–virus.

**Table 1:** Positive and Negative Impact of MB on Viruses

negative effects of the microbiota on viruses			
virus	disease	impact by microbiota	references
rotavirus	diarrhea	administration of *Lactobacillus rhamnosus GG* reduces rotavirus shedding; soluble factor from commensal bacteria inhibits the rotavirus infection	(73)
influenza virus	influenza	higher pulmonary influenza virus titers in antibiotic-treated mice suggest that bacteria (neomycin-sensitive bacteria) harm the virus	(74)
lymphocytic choriomeningitis virus (LCMV)	lymphocytic choriomeningitis	reduced LCMV clearance in antibiotic-treated mice, suggesting that MB promotes antiviral responses	(75)
dengue virus	dengue	MB limits viral replication	(76)
coxsackievirus B3 (CVB3)	cardiac arrhythmias and acute heart failure	MB inhibits CVB3-mediated infection	(77)
murine norovirus (MNV)	gastrointestinal disease	*Lactobacillus* genus can inhibit MNV replication, mediated by the increased expression of IFN-β and IFN-γ	(78)
positive effects of the microbiota on viruses			
poliovirus (PV)	polio	reduced PV replication and pathogenesis in antibiotic-treated mice revealed that MB promotes PV infection	(55)
reovirus	upper respiratory infections, enteritis, fever, and febrile exanthema in childhood	same investigation as of poliovirus (positive impact)	(55)
mouse mammary tumor virus (MMTV)	mammary carcinomas (T cell lymphomas)	MB enhances tolerance to viral via host IL-10 production	(79)
Theiler’s murine encephalomyelitis virus (TMEV)	multiple sclerosis-like diseases	enhanced TMEV replication as well as disease on the administration of lipopolysaccharide (LPS)	(79)

#### 5.1.2 Possible Link between Lungs Microbiome and SARS-CoV-2

SAR-CoV-2 has affected every corner of the world, and new management therapies are in demand to eradicate the pandemic across the globe.^80^ Lungs MB is more dynamic and ephemeral than GIT due to the two-way passage of gases and mucous.^81^ So far, very few studies have explored the microbiome of COVID-19 patients. The modifications of MB in the gut and lung has been linked with COVID-19 patients and have been considered to play a part in altering the immunity and causing more severe complications in COVID-19 patients.^82^ A substantial reduction of MB has been seen in the gut of COVID-19 patients. An overall surge of opportunistic bacteria (e.g., *Rothia*, *Veillonella*, *Streptococcus*, and *Actinomyces*) and reduction of beneficial ones have been seen. Thus, the findings are more inclined to confirm that gut MB interacts with SARS-CoV-2 ^83^.

A study with 106 patients focused on whether post-acute COVID-19 syndrome (PACS) (familiar symptoms include fatigue, reduced memory, and hair loss) is associated with changes in the composition of the gut microbiome. The study found out that the gut microbiome of patients with PACS had increased amounts of *Ruminococcus gnavus* and *Bacteroides vulgatus* while it had decreased amounts of *Faecalibacterium prausnitzii*. The fatigue and reduced memory symptoms were associated with nosocomial gut microbes (e.g., *Clostridium innocuum* and *Actinomyces naeslundii*), while obstinate respiratory symptoms were connected with opportunistic gut pathogens.^84^ This was further supported by another pilot study of 15 COVID-19 patients, where the gut microbiome was identified in the development of opportunistic pathogens (e.g., *Clostridium hathewayi*, *Actinomyces viscosus*, and *Bacteroides nordii*) and reduction of beneficial symbionts (e.g., *Eubacterium ventriosum*, *Faecalibacterium prausnitzii*, *Roseburia*, and *Lachnospiraceae taxa*).^83^ Furthermore, this study revealed that COVID-19 consequences directly depend upon the imbalance of opportunistic pathogens (*Clostridium ramosum* and *Clostridium hathewayi*) and beneficial commensals (e.g., *Alistipes onderdonkii* and *Bacteroides ovatus*). Other than bacterial imbalance, opportunistic fungal growth of *Candida albicans*, *Candida auris*, and *Aspergillus flavus* were also increased compared with controls. The microbiota level and fungal growth seen in COVID-19 patients are shown in Table 2. The relationship between COVID-19 and disease severity with gut microbiome has recently emerged. The gut microbiome might be considered as a therapeutic for managing COVID-19 in the future.^69^

**Table 2:** Microbiota Level and Fungal Growth in COVID-19 Patients

microbiota	increased	decreased	references
bacteria	*Coprobacillus bacterium*	*Faecalibacterium prausnitzii*	(69, 83, 85−92)
	*Clostridium ramosum*	*Bacteroides massiliensis*	
	*Clostridium hathewayi*	*Bacteroides dorei*	
	*Actinomyces spp.*	*Bacteroides ovatus*	
	*Rothia mucilaginosa*	*Bacteroides thetaiotaomicron*	
	*Veillonella infantium*		
	*Streptococcus pneumoniae*		
	*Streptococcus infantis*		
	*Coriobacteriaceae*	*Proteobacteria*	
	*Enterobacteriaceae*	*Bifidobacteria*	
	*Enterococcus*	*Lactobacillus*	
	*Bacteroides nordii*	*Clostridium butyricum*	
	*Streptococcus infantis*	*Clostridium leptum*	
	*Morganella morganii*	*Eubacterium rectale*	
	*Collinsella aerofaciens*	*Lachnospiraceae*	
	*Collinsella tanakaei*	*Roseburia*	
fungi	*Candida spp.*	*Candida parapsilosis*	(93, 94)
	(a) *Candida albicans*		
	(b) *Candida auris*		
	(c) *Candida glabrata*		
	(d) *Candida tropicalis*		
	*Aspergillus spp.*	*Talaromyces wortmannii*	
	(a) *Aspergillus niger*		
	(b) *Aspergillus flavus*		
	(c) *Aspergillus fumigatus*		
	Other genera	*Basidiomycota*	(95, 96)
	(a) *Lophodermium*	(a) *Malassezia yamatoensis*	
	(b) *Aureobasidium*	(b) *Rhodotorula mucilaginosa*	
	(c) *Ascomycota*	(c) *Moesziomyces aphidis*	
	(d) *Debaryomyces*	(d) *Trechispora sp.*	
	(e) *Fusarium*	(e) *Wallemia sebi*	
	(f) *Mucoromycota*		

#### 5.1.3 Bidirectional Gut–Lung Axis: A Pre-COVID-19 Appraisal

A change in gut MB is linked to a bidirectional shift in the interaction among the gut with several essential human organs, which can lead to severe disease symptoms. Except for the lungs, our gut microbiome cluster recently evaluated the bidirectional interaction system among gut microorganisms in addition to key human organs.^97^ Changes in the microbial community of the lungs, including the airways, affect the make-up of the intestinal MB. IBD patients with recognized modifications in their intestinal MB components developed impaired normal functioning of the lungs; for example, roughly half of IBD patients with known abnormalities in their intestinal MB composition have impaired lung function. As a result, the “gut-lung axis” has been proposed as a bidirectional interaction system in which numerous respiratory diseases are frequently tied with gastrointestinal indications.

**Figure 2:**
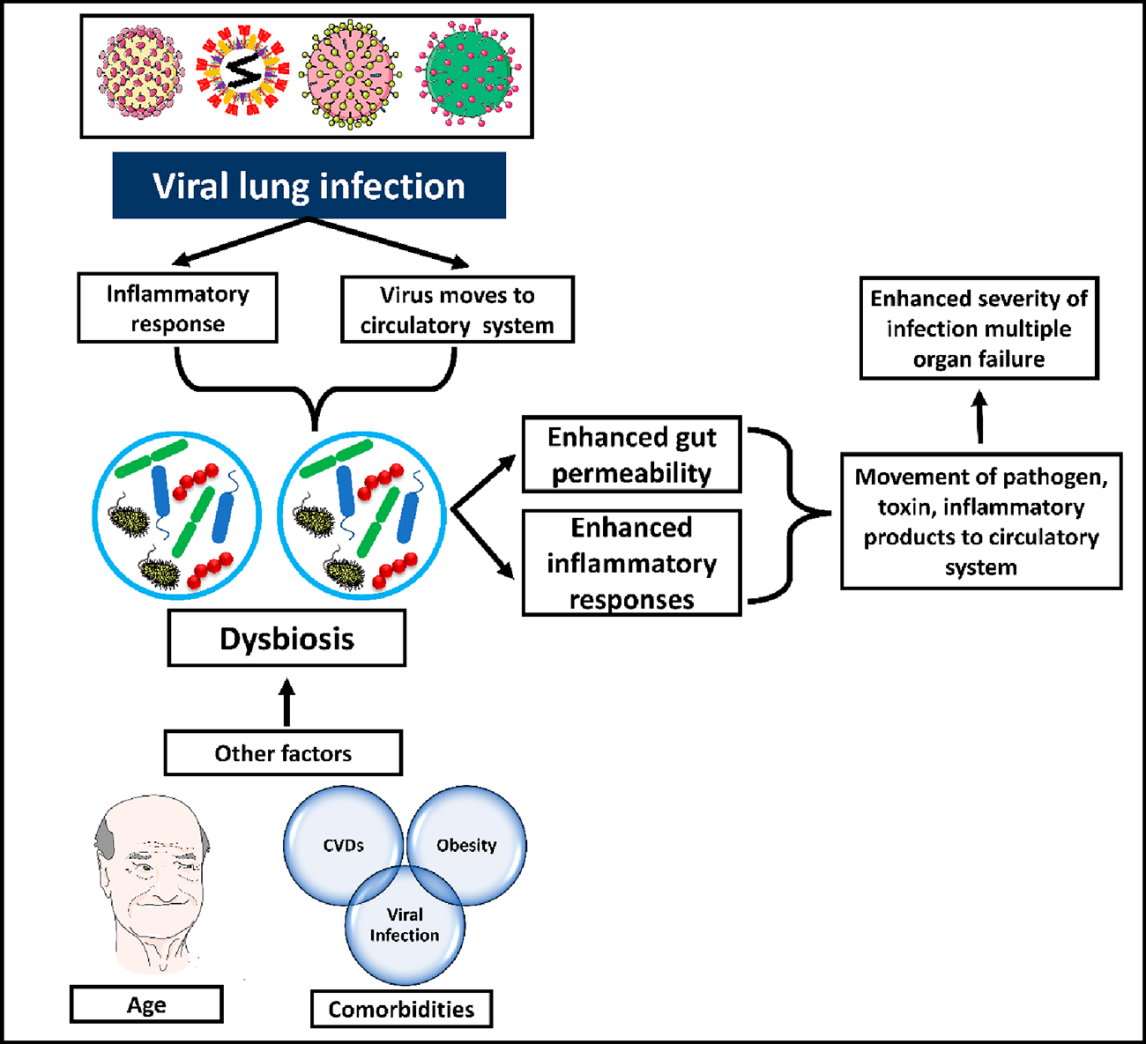
Bidirectional gut-lung axis.

In animal models, for example, *Pneumocystis murina*, *influenza* virus infection, or intratracheal instillation of LPS causes changes in their gut MB.^98^ In mice models, *influenza* virus infection increases *Enterobacteriaceae* while decreasing *Lactococci* and *Lactobacilli* in the gut microbiome. Furthermore, when LPS is administered to mice, the dysbiosis in their airway MB disrupts their gut MB via bacterial translocation beginning from the lungs and ending in the blood. *P. aeruginosa*- or multidrug-resistant *S. aureus*-induced pneumonia is thought to cause gut damage, as *P. aeruginosa*-induced pneumonia causes reduced intestinal epithelial proliferation. Furthermore, mild lung injury interrupts the airway MB, promotes temporary bacterial translocation into the circulation, and results in an acutely elevated bacterial load in the cecum. Patients with COPD have an excessive occurrence of IBD and have intestinal hyper-permeability. The healthy MB, in contrast, maintains tolerogenic immunomodulatory activities in the gut and defends counter to systemic inflammatory disorders.^99^ However, dysbiosis in the intestinal MB has been associated with respirational problems and illnesses. For example, a rise in *Clostridia* and a decrease in *Bifidobacteria* in the stomach have been associated with childhood asthma.^100^ Furthermore, the “gut–lung axis” involves the migration of immune cells from the gut to the respiratory system via circulation, which enhances the host’s defense immunity. Furthermore, the stomach modulates pulmonary responses via host-acquired inflammatory mediators in the circulation. The increased amounts of these inflammatory mediators found in the serum of individuals with gastrointestinal diseases alter immune responses, which implies another method for assessing the local microenvironment in the lungs.^98^ The bidirectional gut–lung axis is briefly shown in Figure 2.

In addition, respiratory viral infections can affect the gut microbiome, which is an important process for priming innate immune responses against respiratory infections and further determining adaptive immune responses against particular pathogens present in the lungs. In pulmonary viral infections, macrophage response to respiratory viruses depends on the gut bacteria. This shows that the lung and the gut are inextricably connected body parts that influence each other’s homeostasis through immunological coordination. Among other ecological factors, microbes are essential in determining normal and diseased immune responses in the lungs and the stomach.^101^ In COVID-19 cases, there is parallel cross-talk among the intestines and the lungs.

The microbial composition of COVID-19 patients’ bronchoalveolar lavage fluid samples is conquered by bacteria often discovered in the mouth and URT.^102^ This was identical to community-acquired pneumonia patients. The microbial fingerprints in the lungs may be used to predict the most prevalent consequence of COVID-19, ARDS, and long-term effects of SARS-CoV-2 outbreaks. The gut MB appears to play a role in boosting antiviral immunity.^103^ As a result, several studies suggest that gut MB manipulation is important in lowering enteritis and ventilator-associated pneumonia and in reversing adverse antibiotic effects to prevent influenza virus replication in the lung epithelium. Though there is currently no clinical indication of gut MB modulation as a COVID-19 treatment, few articles venture into the potential of targeting gut MB as a novel beneficial or adjuvant treatment. Thus, probiotics can improve GI symptoms by maintaining the balance of gut MB and defending the respiratory system by avoiding secondary bacterial infections, which implies the importance of gut MB in ongoing COVID-19 disease.^104^

### 5.2 Gut Microbiome in SARS-CoV-2 Subjects

#### 5.2.1 Viral Interaction with MB

The positive and negative impacts of MBs on the virus are well established.^79^ There is an interaction between bacterial surfaces and the proteins associated with the virus. The interaction between key bacterial envelope components, LPS in the Gram-negative and peptidoglycan (PG) in the Gram-positive, mediates the viral infection. Many viruses, including poliovirus, reovirus, mouse mammary tumor virus, and murine norovirus, have exhibited both LPS and PG to improve receptor affinity, thermostability, and more or less similar pathways to mediate *in vivo* infection.^79^ Collectively, these results indicate the importance of commensal bacteria in enhancing the adherence of the virus to specific proteins, stability, and virulence towards the eukaryotic cells. On the contrary, protection is conferred by MB by facilitating an immunological response to prevent viral contagion. There is a direct and indirect effect of MB on viral biology, and thereby, eukaryotic viruses will alter the biology of the bacteria.^105^

The beginning of MERS-CoV (Middle Eastern respiratory syndrome coronavirus) and SARS-CoV took place in the enteric system of the bat by virtue of commensal bacteria.^106^ The exploitation of the bacteria by strains of SARS-CoV during their emergence amplifies the contagion since the respiratory tract harbours a good amount of commensals.^107^ The onset of augmented disease resulted from the absence of binding of LPS to TLR, as the TLR pathway provides immunity to SARS-CoV. A study indicated the association of the components in the bacterial surface with corona contagion and that the peptidoglycan of the *Bacillus subtilis* decreased the virulence of the coronavirus. The inhibition of the virus due to the presence of surfactin [a cyclic lipopeptide (CLP)] produces temperature- and dose-dependent antiviral characteristics. Moreover, surfactin can reduce the contagion of various enveloped viruses, including Ebola, Zika, Dugbe, influenza, Nipah, Crimean-Congo haemorrhagic fever, chikungunya, Mayaro, and Una viruses.^108^ These reports are sufficient to indicate the role of commensal bacteria in altering the viral contagion, which indicates the crucial role of MB in the pathogenesis of viral infection and various therapeutic options.

Alternate therapeutic options countering the infection caused by MERS-CoV include antimicrobial peptides (AMPs). More than 140 peptides in clinical trials have shown promising results in neutralizing potential viral pathogens, including MERS-CoV.^109^ These peptides inhibit the protein–protein interaction for diseases that are difficult to target. The major advantages offered by these peptide inhibitors are minimal side effects and enhanced specificity. Moreover, various studies have shown that many peptide inhibitors efficiently counteract the viruses.^110^ The mechanisms involved in the antiviral action are virolysis, host cell receptor blockade, inducing an adaptive immune response, and fusion of virus and replication. Peptides with anticorona viral properties inhibit the fusion of the host cell with the virus, which can act on RBD (receptor binding domain) interaction and inhibit HR-1 and HR-2 (heptad repeat) from producing a fusion-active core or S protein cleavage and peptides that inhibit the entry of the virus and further replication. Another possible target could be peptides that inhibit the assembly and release of the virus. The microbial alterations in the fecal matter of 15 COVID-19 subjects revealed an important link with the severity of the disease.^111^ There is a correlation between a highly severe and high baseline of *Coprobacillus*, *Clostridium ramosum*, and *Clostridium hathewayi* and low *Faecalibacterium prausnitzii* and *Alistipes onderdonkii* levels. The enrichment of opportunistic and positive symbiotic bacteria was prevented when exposed to antibiotics.

The fecal samples of 12 patients were found positive when analyzed for the presence of the virus. Out of these, six were still positive at the time of discharge from the hospital. With time, 14 species of bacteria were associated with fecal viral load. These include *Bacteroides massiliensis, Bacteroides thetaiotaomicron*, and *Bacteroides dorei*. Downregulation of the expression of ACE-2 in the murine gut was mediated by *Bacteroides ovatus*, which displayed an inverse correlation, whereas a positive correlation was shown by *Erysipelotrichaceae bacterium2_2_44A*.^69^ The severity of COVID-19 is influenced by the intestinal MB, and the composition of MB and its health play a crucial role in combatting the disease.

### 5.3 Influence of Medications and Polypharmacy on MB and COVID-19

**Figure 3:**
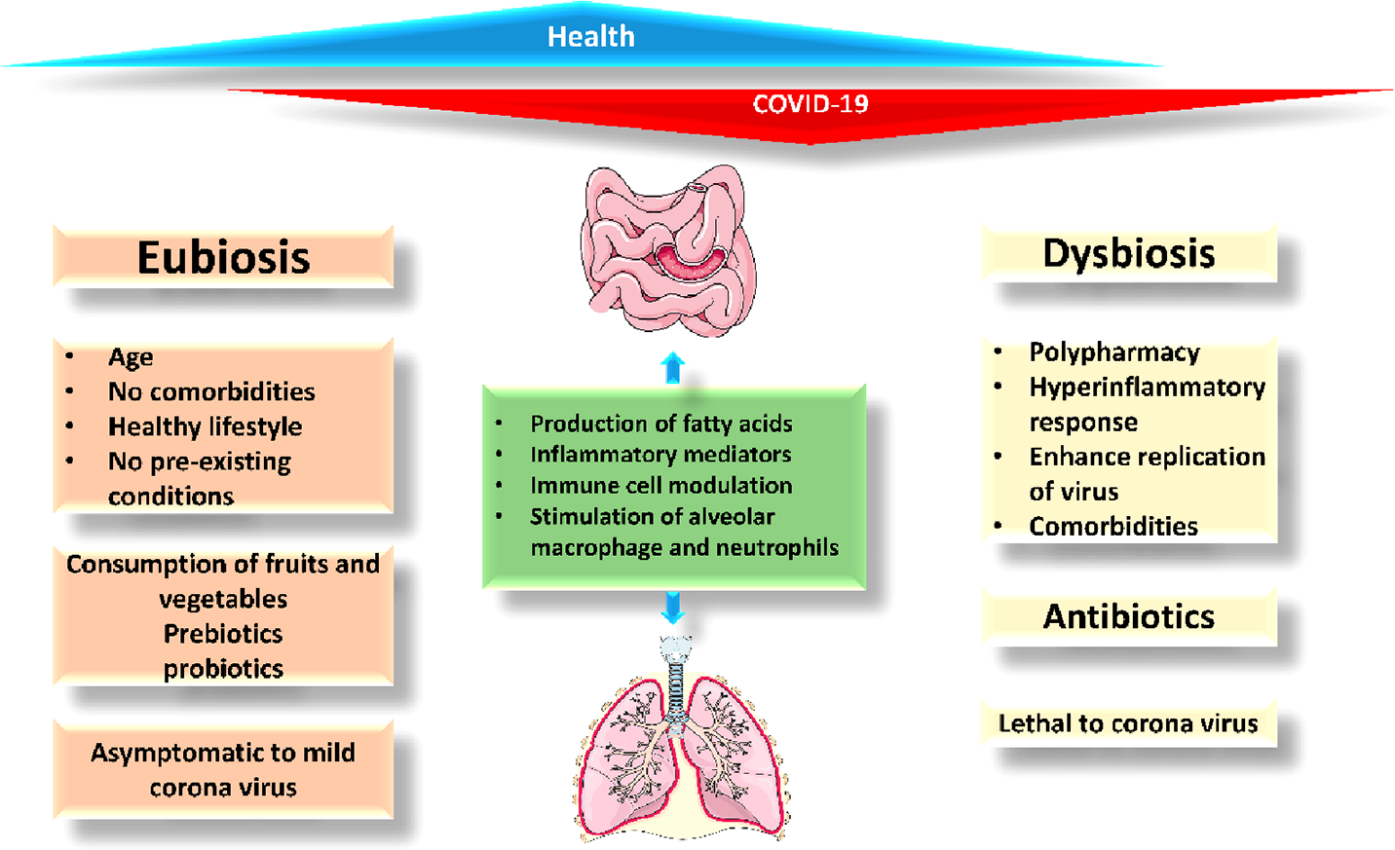
Comparative relation between eubiosis and dysbiosis.

A large proportion of COVID-19 patients are geriatric. Various age-related diseases occur in the elders and render them more vulnerable to COVID-19. One or more than one comorbid conditions, like high blood pressure, a compromised cardiovascular system, hyperlipidaemias, diabetes, and tumor, cause an enhanced risk of death in patients suffering from COVID-19.^112,113^ These abovementioned chronic diseases require a complex therapeutic regimen referred to as polypharmacy (administration of five or more medicaments in a day). The proportional increase in the number of drugs administered implies increased adverse drug reactions and obnoxious effects, which influences the integrity of MB and worsens the host’s capacity to counter viruses, including SARS-CoV-2.^114^ Studies propose that multiple medications have a profound effect on the microbiome’s composition, and the higher the number of coadministered medications, the more the MB will be altered.^114^ It was found that the composition of the microbiome of the aged population and patients with comorbidities were characterized by a relatively higher proportion of pathogenic bacteria like *Helicobacter pylori*, which mediate extragastric pathological complications, and a decrease in *Lachnospiraceae* and *Succinivibrionaceae*, which maintain the pulmonary health of the host and regulate inflammatory processes. The aggravation of the imbalances between pathogenic and symbiotic bacteria represents a life-debilitating condition of COVID-19.^115,116^ The combined relationship between dysbiosis and eubiosis has been studied, and comparison between them leads to understanding how COVID-19 inflicts dysbiosis and imbalances eubiosis, as shown in Figure 3.

#### 5.3.1 MB and Medicines Used to Treat COVID-19

Commonly prescribed drugs used to treat mild COVID-19 are to be administered with pre-existing polypharmacy, which causes a complicated range of interactions with the microbiome. To elucidate further, nonsteroidal anti-inflammatory drugs (NSAIDS) mediate gastrotoxicity by alterations in the MB.^117^ Paracetamol is considered a safer alternative and is widely consumed in COVID-19. Although paracetamol does not alter the microbiome’s composition, there is a drastic increase in absorption and bioavailability in patients with dysbiosis,^118^ which in turn leads to higher vulnerability to liver toxicity and depletion of glutathione. These events exacerbate COVID-19 infection.^115^ Antibiotics, especially broad-spectrum agents prescribed to mitigate/treat superinfections, may profoundly affect the MB of COVID-19 patients. Importantly, azithromycin is a widely used drug for treating COVID-19 because it significantly reduces the bacterial richness (23%), with the composition of MB altered primarily in the *Actinobacteria phylum*. Drop-in genus *Bifidobacterium* was also observed. Therefore, this antibiotic may drastically deplete the already weakened MB status of comorbid COVID-19-positive patients and the elderly.^119^ Antivirals and antibiotics have been given in combination and resulted in more effective treatment. All beneficial therapies, including antiviral, antibiotics, anticoagulants, analgesics, and other adjunctives including vitamin D3 and zinc, have been studied lately.^80^

Vaccines have been developed to end the pandemic, as the spike proteins of SARS-CoV-2 have mostly been targeted to design the vaccine. Various mutants of SARS-CoV-2 have been coming up recently, and vaccines seem to be the only cure.^120,121^ Other than targeting spike proteins, non-spike proteins have also been targeted, including the nucleocapsid protein and membrane protein.^122−124^

One of the most effective drugs administered to reverse hyperinflammation are glucocorticoids. Studies have reported alterations in the gut microbiome in mice^125^ and humans^126^ induced by glucocorticoids. Prednisone was administered for three months to patients diagnosed with acute transverse myelitis. Enrichment in *Firmicutes* and depletion of *Bacteroidetes* were reported in such patients.^126^ As far as antiviral drugs are concerned, remdesivir does not alter the microbiome. In contrast, hydroxychloroquine is no longer recommended in SARS-CoV-2; however, when administered concurrently with doxycycline, the treatment resulted in notable changes in the composition of MB.^127^ Alterations induced by polypharmacy in the gut MB may represent a different but disregarded factor that worsens the compromised microbiome composition in comorbid subjects.^128^

Few suggestions believe consuming a high fiber diet, prebiotics, and probiotics can manipulate the gut MB. Doing so could reduce inflammation, maintain a healthy gut microbiome, and build immune systems.^129^ A lot of experimental research evaluates the effects of prebiotics, probiotics, synbiotics, and postbiotics administration in reducing the severity, duration, and incidence of many viral infections in human beings. The use of probiotic use has not been ruled out, as it is strongly supported by experimental and clinical trials on viruses like rhinovirus, influenza virus, and respiratory syncytial virus.^130^ Successful clinical trials have supported the ongoing discussion of using probiotics as therapeutics in COVID-19. One trial concluded that COVID-19 causes the MB shifts in the gut and upper airway. Application of the probiotics therapy as an adjunctive serves to resolve the severe symptoms of COVID-19 patients, and partial recovery of the dysbiosis associated with COVID-19 was seen.^131^ Many microbes are considered potential candidates and are being used to reduce the severity brought by COVID-19. Mostly microbial adjunctive therapies are being suggested to show effect, e.g., *Lactobacillus rhamnosus, Lactobacillus brevis, Lactobacillus bulgaricus, Lactobacillus casei, Bacillus subtilis, Bifidobacterium longum, Bifidobacterium breve*, *Leuconostoc mesenteroides*, etc.^132^

Many clinical trials have been registered to check the effect of different microbes on the severity of COVID-19. The microbes including *Lactobacillus coryniformis* (K8), *Lactococcus lactis* (W136), *Lactobacillus plantarum* (CECT7481), *Lactobacillus plantarum* (CECT7484), *Lactobacillus plantarum* (CECT7485), and *Pediococcus acidilactici* (CECT7483) are currently in clinical trials to study effects like reducing the severity and effect of probiotics on COVID-19.^133^ Still, no probiotics have been approved by the FDA despite seeing the beneficial effects of many probiotics in relation to COVID-19. The current scenario of using probiotics is only limited to adjunctive treatment, which acknowledges the fact that more research is needed to assert the use of probiotics to improve dysbiosis and inflict eubiosis.^134^

### 5.4 Efficacy of Probiotics in Reducing Duration and Symptoms of COVID-19

The SARS-CoV-2 infection changed fecal microbiomes, which reduced the beneficial microbiomes and increased opportunistic pathogens. The richness of *Coprobacillus* and some *Clostridium* species are linked to the COVID-19 severity. In contrast, the *Faecalibacterium prausnitzii* (anti-inflammatory bacteria) level was inversely linked with the severity of COVID-19, which suggests the impact of SARS-CoV-2 on gut microbiota.^69^ Similarly, other studies support alterations in the gut microbiome and dysbiosis in COVID-19 patients linked with the severity of the disease. This suggests that the administration of beneficial bacteria (probiotics) might provide a beneficial outcome in COVID-19 patients. Probiotics are microbiotic products that can maintain the intestinal flora’s architecture, inhibit harmful microbes, and increase immune response.^135^ Several probiotics are being investigated for their therapeutic potential; *Lactobacillus, Bifidobacteria, Escherichia coli*, and *Enterococcus* are most extensively researched.^136^ Although the probiotics’ mechanism profoundly focuses on the GIT, the effect of probiotics is not confined to the initial infection site. A systemic review compiled the antiviral efficacy of probiotics, and >20 strains have been shown to increase anti-inflammatory interleukins production and immune response.^137^ Moreover, probiotic administration lowered the viral loads directly via immune modulation against SARS-CoV-2 and reduced the secondary infection risk due to COVID-19.^138^ The National Health Commission of China endorsed the use of probiotics for severe COVID-19 patients to restore intestinal balance as well as protect them from secondary infections.^139^ Probiotics such as *Bifidobacterium* and *Lactobacillus* showed a decrease in the common cold, a symptom of SARS-CoV-2.^140^ In a study, probiotic supplementation decreased the disease severity or reduced the disease duration in 2–47% of COVID-19 patients who required ventilation due to ARDS. Probiotics also reduced ventilator-associated pneumonia (VAP) and shortened the antibiotic duration for VAP.^141^

COVID-19 is associated with several organs and, thus, requires an anti-inflammatory approach to control systemic inflammation^113^ and to reduce death and severity of COVID-19 patients due to “cytokine storm”. The administration of probiotics increases survival and reduces the viral load and bronchial epithelial damage in influenza and pneumonia, mediated by immunomodulation, virucidal activity by recruiting of NK lymphocytes, alveolar macrophages, and increased proinflammatory mediators(IL-6 and TNF-α) in the early phase and increased anti-inflammatory mediators, like IL-10 and Treg cells, in lungs in the late phase to reduce lung injury.^142,143^ Moreover, various probiotics in the prevention of respiratory diseases are well reviewed by Bottari et al.^144^ These studies suggest the anti-inflammatory potential of probiotics. Therefore, probiotics with anti-inflammatory potential can be used to hamper the COVID-19-associated secondary infection. Currently, the efficacy of various probiotics in reducing the duration and symptoms of COVID-19 (PROVID-19) are under clinical trial for different strains, as summarized in Table 3. Probiotics might open therapeutic avenues for the reduction in duration of infection and associated symptoms of COVID-19.

**Table 3:** Clinical Trials Study of Probiotics in COVID-19

sr. no.	probiotics	sponsor (organization)	estimated number of participants	duration of intervention	clinical trial phase	current status	NCT
1	*Lactobacillus rhamnosus GG*	Duke University	182	daily 2 capsules for 28 days	interventional	recruitment completed	NCT04399252
2	*Lactobacillus plantarum* 299v	Medical College of Wisconsin	80	daily for 8 weeks	interventional	recruiting	NCT05227170
3	*Ligilactobacillus salivarius* MP101	Universidad Complutense de Madrid	25	daily for 4 months	interventional	recruiting	NCT04922918
4	Omni-Biotic Pro Vi 5	King Edward Medical University	50	daily 2 tablets for 14 days	interventional	recruitment completed	NCT05043376
5	BLIS K12	Medical University of Graz	20		interventional	recruiting	NCT04813718
6	*L. reuteri* DSM 17938 + vitamin D	Örebro University, Sweden	161	daily 2 capsules for 6 months	interventional	recruitment completed	NCT04734886
7	*Lactobacillus salivarius*	ProbiSearch SL	41	daily 1 capsule for 28 days	interventional	recruitment completed	NCT04937556
8	probiotics (2 strains)	Centre de recherche du Centre hospitalier universitaire de Sherbrooke	17	daily for 25 days	interventional	recruitment completed	NCT04621071
9	probiotics	Centre de recherche du Centre hospitalier universitaire de Sherbrooke	618	daily for 25 days	interventional	recruiting	NCT05080244
10	Symprove	King’s College Hospital NHS Trust	60		interventional	not yet recruiting	NCT04877704
11	probiotics	Bioithas SL	41	daily 1 capsule for 30 days	interventional	recruitment completed	NCT04390477
12	probiotics	Hospital de Sagunto	96		interventional	recruiting	NCT04666116
13	Probiorinse (*Lactococcus lactis W136*)	Centre hospitalier de l’Université de Montréal (CHUM)	23	twice daily for 14 days	interventional	recruitment completed	NCT04458519
14	microbiome immunity formula	Chinese University of Hong Kong	280	daily for 3 months	interventional	recruiting	NCT04950803
15	*bidido-* and *lactobacteria*	Nordic Biotic Sp. z o.o.	100	daily for 28 days	interventional	recruiting	NCT04907877
16	ABBC1 immunoessential	AB Biotek	90	30 days	interventional	recruiting	NCT04798677

### 5.5 Clinical Studies of Probiotics against COVID-19

Microbiota constitutes an important part of the human body and it has a pivotal role in normal homeostasis. The human body is the reservoir of the microbes that outnumber the total human cells occupying skin and the mucosal membranes. The gut microbiota has a crucial role in the maintenance of immunity. Many studies have reported that germ-free mice have shown protective effects of the microbiome in diseased conditions like metabolic, inflammatory, and infectious diseases.^145−147^

It has been reported in a pilot study that there was a significant decrease in the beneficial commensals and increases in the pathogenic microbes in fecal samples of 15 patients.^69^ COVID-19 severity has been correlated with the baseline abundance of *Clostridium* and *Coprobacillus* species.^136^ The inverse correlation of COVID-19 severity was reported with the abundance of *Faecalibacterium prausnitzii*, an anti-inflammatory bacterium showing the SARS-CoV-2 influence on microbiota.^69^ Another pair of studies by Liu et al. and Yeoh et al. showed a significant change in the microbiome and dysbiosis in the subjects suffering from SARS-CoV-2, associated with inflammatory markers and disease severity.^148,149^

It was suggested by a research group that the noninfected healthy subjects’ microbiome is highly predictive of the blood proteomic biomarkers of SARS-CoV-2 disease severity.^150^ Dysbiosis of the gut microbiota increases the risk of abnormal inflammatory states like increased susceptibility and SARS-CoV-2 infection severity. The commensal probiotic strains may improve the lung and gut through their SCFAs and host-mediated chemokines and cytokines.^147^ The microbiome can immunomodulate an individual’s immune system, so probiotics can be expected to improve the health and immune response against infections like SARS-CoV-2. Present evidence demonstrates an association of the microbiome with COVID-19 susceptibility and severity. Several studies have investigated that probiotics have demonstrated preventive effects against SARS-CoV-2 infections.^69,84,148,149,151^

Few have put forward the indirect evidence of probiotic association with SARS-CoV-2 infectivity on the basis of previous SARS-CoV infections.^136^ Many studies have demonstrated the efficacy of probiotics against viral infections. It has been shown that around 20 strains have improved the anti-inflammatory cytokines and antibody production against viruses.^136^ Probiotic supplementation has also been reported to decrease the SARS-CoV-2 viral load by modulating the immune system and fighting against COVID-19 and other infections.^152^ Several countries like China recommended using probiotics in SARS-CoV-2-affected patients to restore the microbiome and prevent them from secondary infections.^136^ Therefore, with the growing shreds of evidence, it is clear that the microbiome is enhancing immunity, thereby suggesting a preventative strategy against infections like SARS-CoV-2.

## 6 Future Perspectives

SARS-CoV-2 primarily affects the lungs via interaction with the ACE-2 receptor.^121^ Interestingly, SARS-CoV-2 RNA was also detected in the feces of COVID-19 patients,^153,154^ which suggests its impact on the GI tract. Studies suggest that SARS-CoV-2 susceptibility might be related to microbiome composition among the diverse cohorts.^151^ Investigations of fecal samples revealed that amino acid pathways might be involved between COVID-19 susceptibility and microbiome composition. The microbiome and its metabolites may protect the susceptible population against infectious diseases like SARS-CoV-2.^99^ So, it is important to decipher such amino acid pathways to achieve a better understanding. There are immense requirements to find out microbiota metabolites, particularly how they immunomodulate to protect against infectious disease. It will be great to see the cross-talk of proteins in the human body with the microbiota to provide protection. For survival, there is a requirement for appropriate systemic inflammatory control because COVID-19 is a multiorgan phenomenon. The cytokine storm is the major reason for COVID-19-related deaths, and treatment of this hyperinflammatory state in COVID-19 subjects may be a good strategy for its treatment and management.^155^ In this regard, probiotics might prove as a potential treatment option. Several preclinical investigations have focused on pneumonia and influenza and shown advantages from the administration of probiotics that enhanced survival, reduced viral load, and weight loss.^142,143,156,157^ The probiotics should be used at the clinical level to restore the original microbiome of virus-infected patients. A study also reported that probiotics could modulate vitamin D and maintain the growth and composition of the microbiome.^99^ All these benefits of probiotics make them suitable candidates for managing infectious diseases. However, studies must consider larger cohorts, a wide age group, longer durations, and geographical locations in COVID-19 patients to reach conclusive results on identifying alterations in microbiota composition, which subsequently affects the gut–lung axis during SARS-CoV-2 infection. These research results will provide more convincing evidence of the microbiota’s function in COVID-19 disease and enable the development of improved management techniques that can be used to treat COVID-19.

## 7 Conclusions

We have given the current overview of how microbiome alteration affects disease severity concerning SARS-CoV-2. It was observed that the composition and health of the gut and lung MB play a pivotal role in the progression and worsening of COVID-19 infection. Any changes to the composition of MB caused by medications or pre-existing comorbidities influence the complications associated with the infection. Dysbiosis results in enhanced gut permeability and cytokine storm, which ultimately magnifies the severity of COVID-19 by translocating pathogenic microorganisms, toxins, and inflammatory products to the circulatory system. It is clear from the preclinical and clinical data that the microbiome plays a crucial role, and the design of a particular strain of probiotics can be a game-changing approach against any infection. Probiotics have proved and demonstrated protection against COVID-19 infection in preclinical and clinical settings. The complexities of MB in the gut and lung are far more than being investigated. Further studies are required to reach any definitive conclusion. The present review affirms the overwhelming evidence that SARS-CoV-2 infection is accompanied by major changes in the gut/airway microbiota composition, which affects the course and prognosis of the disease.

## Abbreviations

ACE-2: angiotensin-converting enzyme-2
AGE: acute viral gastroenteritis
ARDS: acute respiratory distress syndrome;
AMPs: antimicrobial peptides;
ATM: antibiotic-treated mice
CIT: culture-independent techniques
COVID-19: coronavirus disease 2019
CF: cystic fibrosis
COPD: chronic obstructive pulmonary disease
CVB3: coxsackievirus B3
DC: dendritic cell
GALT: gut-associated lymphoid tissues
GFM: germ-free mice
GI: gastrointestinal
GM-CSF: granulocyte-macrophage colony-stimulating factor
IBD: inflammatory bowel disease
IL: interleukin
LCMV: lymphocytic choriomeningitis virus
LPS: lipopolysaccharide
LRT: lower respiratory tract
MALT: mucosa-associated lymphoid tissue
MHC: major histocompatibility complex
MB: microbiota
MERS-CoV: Middle Eastern respiratory syndrome coronavirus
MMTV: mouse mammary tumor virus
MNV: murine norovirus
NSAIDS: nonsteroidal anti-inflammatory drugs
OPs: opportunistic pathogens
PG: peptidoglycan
PV: poliovirus
SARS-CoV-2: severe acute respiratory syndrome coronavirus 2
TGF-β: transforming growth factor-beta
Th cells: helper T cells
TLR: toll-like receptors
TMEV: Theiler’s murine encephalomyelitis virus
PACS: post-acute COVID-19 syndrome
PRRs: pattern recognition receptors
Treg: regulatory T cells
UC: ulcerative colitis
URT: upper respiratory tract
VAP: ventilator-associated pneumonia
VCs: viral clusters
